# Association of Abdominal Obesity with Lumbar Disc Degeneration – A Magnetic Resonance Imaging Study

**DOI:** 10.1371/journal.pone.0056244

**Published:** 2013-02-13

**Authors:** Jani Takatalo, Jaro Karppinen, Simo Taimela, Jaakko Niinimäki, Jaana Laitinen, Roberto Blanco Sequeiros, Dino Samartzis, Raija Korpelainen, Simo Näyhä, Jouko Remes, Osmo Tervonen

**Affiliations:** 1 Institute of Clinical Medicine, University of Oulu, Oulu, Finland; 2 Finnish Institute of Occupational Health, Oulu, Finland; 3 Oulu University Hospital, Oulu, Finland; 4 Department of Public Health, University of Helsinki, Helsinki, Finland; 5 Institute of Diagnostics, University of Oulu, Oulu, Finland; 6 Department of Orthopaedics & Traumatology, University of Hong Kong, Pokfulam, Hong Kong, Special Administrative Region, People’s Republic of China; 7 Institute of Health Sciences, University of Oulu, Oulu, Finland; 8 Department of Sports and Exercise Medicine, Oulu Deaconess Institute, Oulu, Finland; University of Washington School of Medicine, United States of America

## Abstract

**Purpose:**

To evaluate whether midsagittal (abdominal) obesity in magnetic resonance imaging (MRI), waist circumference (WC) and body fat percentage are associated with lumbar disc degeneration in early adulthood.

**Methods:**

We obtained the lumbar MRI (1.5-T scanner) of 325 females and 233 males at a mean age of 21 years. Lumbar disc degeneration was evaluated using Pfirrmann classification. We analysed the associations of MRI measures of obesity (abdominal diameter (AD), sagittal diameter (SAD), ventral subcutaneous thickness (VST), and dorsal subcutaneous thickness (DST)), WC and body fat percentage with disc degeneration sum scores using ordinal logistic regression.

**Results:**

A total of 155 (48%) females and 147 (63%) males had disc degeneration. AD and SAD were associated with a disc degeneration sum score of ≥3 compared to disc degeneration sum score of 0–2 (OR 1.67; 95% confidence interval (CI) 1.20–2.33 and OR 1.40; 95% CI 1.12–1.75, respectively) among males, but we found no association among females. WC was also associated with disc degeneration among males (OR 1.03 per one cm; 95% CI 1.00–1.05), but not among females.

**Conclusion:**

Measures of abdominal obesity in MRI and waist circumference were associated with disc degeneration among 21-year-old males.

## Introduction

Obesity is a worldwide concern because it increases the risk of various health disorders such as cardiovascular diseases, strokes, diabetes, cancers, metabolic syndrome, non-alcoholic fatty liver, and asthma. It also leads to psychosocial problems, decreases productivity, and adds to health-care costs [1], [2], [3]. As such, obesity is an important public health issue, the prevalence of which is continuously increasing, in the USA [4] and in various parts of Europe in particular [5]. Among Finnish adolescents, the prevalence of overweight has doubled and obesity tripled in the last 20 years [6].

Low back pain (LBP) is a most debilitating condition, and can lead to decreased physical function, compromised quality of life, and psychological distress [7]. Obesity has been recently recognized as a risk factor of LBP [8], [9]. Risk factors associated with cardiovascular disease have also been implicated in the development of LBP [10], [11].

Recent evidence indicates that disc degeneration in magnetic resonance imaging (MRI) is associated with LBP [12]–[14]. Therefore, the aetiology of disc degeneration is clinically relevant. Heritability plays a major role in the development of disc degeneration [15] but genetic factors, at least currently, cannot be modified. Overweight and obesity in turn, for which body mass index (BMI) serves as a proxy measure, are modifiable risk factors. BMI has been implicated in disc degeneration among both adults and adolescents [13], [14], [16].

There are several methods for measuring body composition, including BMI, waist circumference (WC), waist-to-hip ratio, bioelectrical impedance analysis, underwater weighing, dual-energy X-ray absorptiometry (DXA), and magnetic resonance imaging (MRI). Although BMI has been used as a standardized measure to assess overweight and obesity, its limitations include that it cannot account for the distribution of body fat and muscle mass [17]. MRI and DXA have proved to be valid methods for measuring the adiposity of the body [18]. MRI has also proved accurate for measuring abdominal obesity [19]. In fact, abdominal obesity seems to be related to the development of cardiovascular diseases and to be more sensitive to this than BMI [20].

Since the measurement of BMI in relation to disc degeneration has numerous limitations, more sensitive analysis of the body’s true fat distribution, in particular abdominal obesity, is needed. We hypothesize that, rather than increased lean mass, an increased amount of adiposity is associated with lumbar disc degeneration. As such, the present study addresses the assessment of adiposity in MRI and its relationship with lumbar disc degeneration.

## Materials and Methods

### Study Population

**Figure 1:**
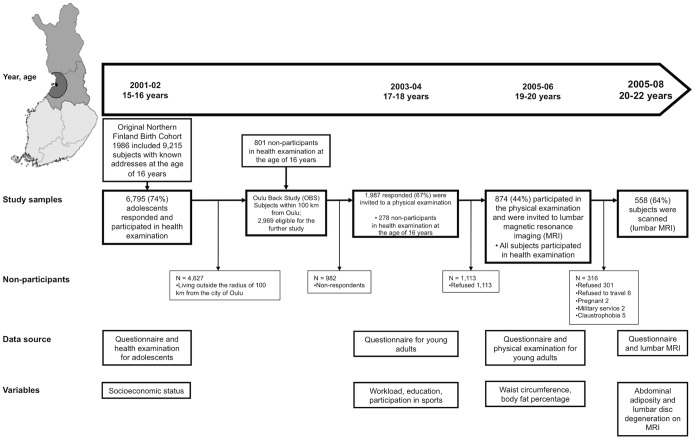
Flow-chart of study population. The Study population consisted of the members of the Northern Finland Birth Cohort 1986 (NFBC 1986) in the two northernmost provinces of Finland (n = 9479) who lived within 100 km of the city of Oulu in 2003 (n = 2969). Those who participated in the physical examination at 19 years of age were invited to lumbar magnetic resonance imaging (MRI), which was performed between November 2005 and February 2008 at a mean participant age of 21 years. LBP = low back pain.

In 2003–2004, when they were approximately 18 years old, a postal questionnaire was sent to all members of the 1986 Northern Finland Birth Cohort (NFBC 1986) living within 100 km of the city of Oulu (n = 2969; Oulu Back Study). The respondents (n = 1987, response rate 67%) were invited to a physical examination in 2005–2006 in which height, weight, WC and body fat percentage were measured and postural, workload, and physical activity factors elicited by a questionnaire. A total of 874 participants (44% of those invited) attended the examination at the mean age of 19 and were further invited to lumbar spine MRI (Fig. 1). A total of 558 (64% of those who participated in the physical examination; 28% of the population of Oulu Back Study) participants attended the MRI examination in 2007–2008, at the average age of 21.

The study population is a subpopulation of the NFBC 1986, which consists of 9479 children with an expected date of birth between July 1, 1985 and June 30, 1986 in the two northernmost provinces of Finland; Oulu and Lapland.

Some differences between the non-participants (n = 2408) and MRI participants (n = 563) have been previously reported [21]. In short, the participants were mostly females, physically more active and more likely to be non-smokers than non-participants, and a higher proportion of them suffered from LBP (Table S1). We also noted that the non-participants had more missing data than the participants.

The Ethics Committee of the Northern Ostrobothnia Hospital District reviewed the study plan and the study was performed according to the Declaration of Helsinki.

### Lumbar Magnetic Resonance Imaging

Participants were scanned using 1.5 T unit equipment (Signa, General Electric, Milwaukee, WI, USA) with a phased array spine coil (USA Instruments, Aurora OH, USA) and two imaging protocols of the entire lumbar spine: a sagittal T1-weighted (440/14 [repetition time msec/echo time msec]) spin echo, and T2-weighted (3960/116) fast spin echo. The slice thickness was 4 mm, with a 1 mm interslice gap. The detailed MRI protocol has been presented elsewhere [21].

We assessed the degree of disc degeneration from T2-weighted images [21] using modified Pfirrmann classifications: Grade 1 (normal shape, no horizontal bands, clear distinction of nucleus and annulus), Grade 2 (non-homogeneous shape with horizontal bands, some blurring between nucleus and annulus), Grade 3 (non-homogeneous shape with blurring between nucleus and annulus, annulus shape still recognizable), Grade 4 (non-homogeneous shape with hypointensity, annulus shape not intact and distinction between nucleus and annulus impossible, disc height usually decreased), and Grade 5 (same as Grade 4 but with collapsed disc space). Grades 1 to 2 were classified as normal discs, while grades 3 to 5 were defined as degenerated. We obtained the sum score of disc degeneration by summing the scores of each lumbar disc. Normal discs (Grades 1 and 2) were scored as 0, and with each higher degree of disc degeneration the score increased by one. The scores of the entire lumbar spine were then summated, according to the individual disc scores. Therefore, the sum score theoretically ranged from 0 to 15 for five lumbar discs (although the actual values ranged from 0 to 8).

Disc degeneration was evaluated by two experienced musculoskeletal radiologists (JN and RB), who were blinded to the participants’ clinical status. The inter-rater reliability was assessed with kappa statistics, which is considered the correct approach to analyzing the agreement in dichotomized (yes vs. no) variables [22].

**Figure 2:**
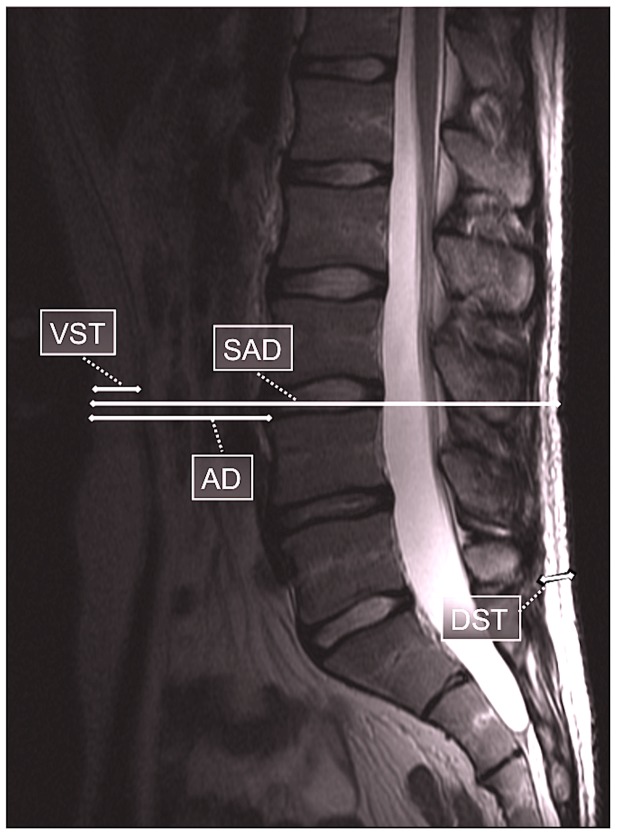
Midsagittal image of lumbar spine showing level of measurement: abdominal diameter (AD), sagittal diameter (SAD), ventral subcutaneous thickness (VST), and dorsal subcutaneous thickness (DST). AA, SAD, and VST are not at the same level in the image as that measured in the study, due to technical reasons.

Four adiposity diameters were measured in the midsagittal slice from T2-weighted images at the level of the lumbar spine: abdominal diameter (AD, cm), sagittal diameter (SAD, cm), ventral subcutaneous thickness (VST, cm), and dorsal subcutaneous thickness (DST, cm) (Fig. 2). We chose the MRI image for measuring the adiposity diameters according to the clearest image of spinal processes and widest cerebrospinal fluid space. The SAD and AD were defined as the narrowest diameter from abdominal subcutaneous fascia to the dorsal subcutaneous fascia and the anterior border of the vertebral body, respectively, at the level of L3 or L4 vertebral body. The VST, presenting subcutaneous adipose tissue, was measured at the same level as SAD and AD. DST was measured perpendicular to the skin at the presacral level, from the subcutaneous fat extending to the subcutaneous fascia between the spinal processes of L5 and S1. The first author assessed all MRI obesity measurements but a musculoskeletal radiologist (JN) assessed 30 (5%) randomly assigned participants for inter-rater reliability.

### Anthropometric Measures

Waist circumference was measured at halfway between the iliac crest and the lowest rib [23] and body fat percentage was assessed using bioelectrical impedance (InBody, Mega Electronics Ltd, Kuopio, Finland). The bioelectrical impedance method measures body composition by sending a safe, low electrical current through the body. The current passes freely through the fluids contained in muscle tissue, but encounters difficulty/resistance when it passes through fat tissue. This resistance of the fat tissue to the current is termed ‘bioelectrical impedance’. The resistance difference between conductors provides the measure of the adipose tissue content of the body. Anthropometric measurements were performed at the age of 19; no anthropometric data were available at 21 years.

### Statistical Analyses

The association of disc degeneration sum score with adiposity measures was first inspected by comparing the mean adiposity measures between disc degeneration classes by the use of 95% confidence intervals (CI) and one-way analysis of variance (ANOVA). We conducted further analyses by ordinal logistic regression based on proportional odds assumption [24]. In this analysis, the outcome was the degree of disc degeneration, measured in ordered classes. The original sum scores (0 to 8) were re-classified to form ordered classes *i = *1 to 4, corresponding to disc degeneration sum scores of 0, 1, 2 and 3–8 (the six highest classes were merged because the figures were so small). The associations between the outcome and explanatory variables were sufficiently linear and were treated as such. The results were expressed as odds ratios (OR) together with their 95% CI. Here, the OR expresses the ratio of odds for having a disc degeneration sum score equal to or higher than this in any given ordinal category *i*, compared with all classes lower than *i*. This method combines the information from all ordered categories under the assumption that the ORs over all pairs of categories ≥ *i* vs.<*i* are similar (proportionality assumption). We used the Wald test to check the proportionality assumption [25]. In instances not fulfilling this assumption (p value <0.05), separate ORs were shown for all levels of *i*. We first entered each explanatory variable into the model alone to produce crude ORs. Then we calculated adjusted ORs by entering postural, workload, and activity variables: heavy physical work, driving a motor vehicle, lifting heavy objects at work, previous injury, socioeconomic status and education. The latter factors were allowed for since they could be related to the outcome and the explanatory variables and may therefore confound the results. We did not adjust for driving a motor vehicle and lifting heavy objects at work among women because the figures were small (one and nine women, respectively). Additional postural, workload, and activity factors were considered (kneeling/squatting at work, hands above the shoulder level at work, awkward trunk postures, using vibrating tool(s), sedentary work, standing or walking at work, and participation in sports), but they did not change the parameter estimates and were thus omitted. All variables regarded as confounders are explained in Table S2. All analyses were stratified by gender. The ORs were obtained by the *gologit2* procedure of the Stata 11 software [26].

Spearman correlations were used to assess the correlation of abdominal obesity with WC and body fat percentage. We analyzed the inter-rater agreement of the disc degeneration and MRI abdominal measurements for two observers using kappa statistics and interclass coefficient correlation (ICC), respectively. Values of >0.80 were considered very good, 0.61–0.80 good, 0.41–0.60 moderate, 0.21–0.40 fair, and ≤0.20 poor for inter-rater repeatability and correlation between adiposity measures [22]. The Chi square test was used to test the differences between genders in the prevalence of disc degeneration at each level and the sum score of disc degeneration.

## Results

We measured the DST of all (n = 558) participants. The VST, AD and SAD of 53 (34 males and 19 females) participants could not be measured, because the subcutaneous fascia was not visible due to obesity. The VST of 43 participants was also immeasurable (28 males and 15 females). However, the AD and SAD values were measured as far anteriorly as possible, and the values were in the highest quartile of both variables. Waist circumference and bioelectrical impedance measurements were available for all males but only for 323 and 321 females, respectively. At least one degenerated disc was observed in 54% of the participants (63% of males and 48% of females). The disc degeneration sum score ranged from 0 to 8 (Table 1). The mean disc degeneration sum score among females was 1.0 (SD 1.4, range 0–8) and among males 1.4 (SD 1.4, range 0–6).

**Table 1:** Distribution of sum scores of disc degeneration (DD) and presence of DD by lumbar level according to gender.

	Females N = 325	Males N = 233	p-value		
DD sum score	% (N)				
0	52.3 (170)	36.9 (86)		0.033*	
1	19.4 (63)	23.2 (54)			
2	14.5 (47)	19.7 (46)			
3	5.8 (19)	10.3 (24)			
4	5.5 (18)	7.3 (17)			
5	1.2 (4)	1.3 (3)			
6	0.9 (3)	1.3 (3)			
7	–	–			
8	0.3 (1)	–			
DD L1/2	6.8 (22)	7.7 (18)		0.216	
DD L2/3	4.6 (15)	3.8 (9)		0.781	
DD L3/4	6.1 (20)	8.2 (19)^a^		0.319	
DD L4/5	19.7 (64)	27.5 (64)		0.003	
DD L5/S1	34.5 (112)	51.9 (121)		<0.001	

* From chi square test (χ2 = 15.17, def = 7).

a 232 disc evaluated in males due to an implant in one participant at L3/4 Percentages (numbers) of participants presented.

The kappa statistics for inter-rater reliability between the radiologists was poor for L1–2 and L2–3 disc degeneration (κ = 0.05 and 0.12, respectively), but moderate to good for the other levels (κ = 0.41, 0.63 and 0.50 for L3–4, L4–5 and L5-S1, respectively). Finally, the two radiologists reviewed all discrepancies by consensus reading. Repeatability of MRI abdominal measurements (ICC) was very good, at 0.85, 0.95, 0.91, and 0.99 for AD, SAD, VST, and DST, respectively.

The correlation of WC with SAD (0.74) and body fat percentage with DST (0.70) were high, whereas a moderate correlation was found between WC (0.59) and AD, and between body fat percentage (0.52) and VST.

The females had lower AD, SAD and WC than the males, while the males had lower VST, DST and body fat percentage than the females. The means and ranges of the MRI obesity measures, WC and body fat percentages are shown in Table 2. WC, SAD and AD were significantly higher among the males whereas VST, DST and body fat percentage were significantly higher among the females.

**Table 2:** Means (ranges) of adiposity MRI, waist circumference and body fat percentage measurements.

	Males	Females	All	Number of valid observations
Abdominal adiposity (cm)	7.2 (3.6–11.6)	6.7 (2.9–11.5)	6.9 (2.9–11.6)	558
Sagittal diameter (cm)	17.5 (13.1–22.9)	16.2 (11.9–24.2)	16.7 (11.9–24.2)	558
Ventral subcutaneous thickness (cm)	1.6 (0.4–5.3)	2.0 (0.4–5.9)	1.9 (0.4–5.9)	515
Dorsal subcutaneous thickness (cm)	1.7 (0.1–6.4)	2.4 (0.4–7.2)	2.1 (0.1–7.2)	558
Waist circumference (cm)	81.8 (62.5–121.0)	72.3 (59.0–118.5)	76.3 (59.0–121.0)	556
Body fat (%)	16.1 (5.5–38.1)	26.5 (11.9–48.2)	22.1 (5.5–48.2)	554

Table 3 compares the means of adiposity measures between disc degeneration classes and shows elevated AD and SAD among men in the highest disc degeneration class. Among women, the trends were similar to those among men, but did not reach statistical significance.

**Table 3:** Means (95% confidence intervals) of adiposity measures, classified by disc degeneration sum score.

	Disc degeneration sum score				p value^a^	No.
	0	1	2	3–8		
*Men*						
Abdominal diameter (mm)	71 (68–74)	68 (64–73)	73 (69–78)	77 (72–81)	0.048	233
Sagittal abdominal diameter (mm)	172 (167–176)	171 (166–177)	175 (169–181)	184 (177–190)	0.012	233
Ventral subcutaneous thickness (mm)	16 (14–18)	16 (14–19)	17 (14–19)	17 (15–20)	0.834	205
Dorsal subcutaneous thickness (mm)	16 (13–18)	17 (15–20)	17 (14–20)	19 (16–22)	0.300	233
Waist circumference (mm)	80 (78–82)	83 (80–85)	83 (80–85)	84 (81–86)	0.106	233
Body fat percentage	16 (14–17)	16 (15–18)	16 (14–18)	16 (15–18)	0.940	233
*Women*						
Abdominal diameter (mm)	68 (65–70)	65 (61–69)	64 (59–69)	69 (64–74)	0.304	325
Sagittal abdominal diameter (mm)	163 (159–166)	161 (156–167)	158 (152–165)	166 (160–173)	0.357	325
Ventral subcutaneous thickness (mm)	20 (19–21)	21 (19–24)	18 (15–21)	22 (19–25)	0.151	310
Dorsal subcutaneous thickness (mm)	24 (23–26)	23 (21–26)	23 (19–26)	25 (21–28)	0.803	325
Waist circumference (cm)	73 (71–74)	71 (69–73)	72 (69–74)	74 (71–76)	0.286	323
Body fat percentage	27 (26–28)	26 (24–27)	25 (23–27)	27 (25–29)	0.186	321

a From one-way ANOVA.

Ordinal logistic regression showed significant associations of disc degeneration with AD and SAD, and a weak association with WC, but among males only (Table 4). According to crude analyses, the odds for having disc degeneration increased by 17%, 16% and 3% per one-centimeter increase of AD, SAD and WC, respectively, at all levels of disc degeneration. When potential confounders were taken into account, AD and SAD did not meet the proportionality assumption. The separate ORs for each level of the outcome revealed no increase in disc degeneration at its lowest level (class 1 vs. 0) but did show significant increases at two higher levels of disc degeneration (50% and 67% for AD; 24% and 40% for SAD). For WC, the OR remained unchanged after adjustment; only its confidence interval was marginally wider. Disc degeneration was not significantly associated with VST, DST or body fat percentage, and we found no associations at all among females.

**Table 4:** Odds ratios (OR) and their 95% confidence intervals (CI) for crude and adjusted ordinal logistic regression analyses on association between lumbar disc degeneration (DD) sum score (in four ordinal classes) and measures of abdominal obesity.

Explanatory variable	Males		Females	
	Crude OR (95% CI)	Adjusted^a^ OR (95% CI)	Crude OR (95% CI)	Adjusted^b^ OR (95% CI)
Abdominal diameter (cm)	1.17 (1.01–1.36)		0.96 (0.85–1.09)	0.94 (0.81–1.09)
Sum score 1 vs. 0		0.78 (0.60–1.01)		
Sum score 2 vs. 0–1		1.50 (1.13–1.98)		
Sum score 3–8 vs. 0–2		1.67 (1.20–2.33)		
No. of observations	233	151	325	225
Sagittal abdominal diameter (cm)	1.16 (1.04–1.30)		1.00 (0.91–1.09)	0.98 (0.88–1.09)
Sum score 1 vs. 0		0.97 (0.82–1.15)		
Sum score 2 vs. 0–1		1.24 (1.04–1.49)		
Sum score 3–8 vs. 0–2		1.40 (1.12–1.75)		
No. of observations	233	151	325	225
Ventral subcutaneous thickness (cm)	1.13 (0.85–1.51)	0.85 (0.56–1.29)		1.00 (0.76–1.32)
Sum score 1 vs. 0			1.06 (0.84–1.35)	
Sum score 2 vs. 0–1			0.95 (0.72–1.24)	
Sum score 3–8 vs. 0–2			1.28 (0.92–1.77)	
No. of observations	205	137	310	210
Dorsal subcutaneous thickness (cm)	1.21 (0.97–1.51)	1.11 (0.81–1.51)	0.96 (0.81–1.15)	0.98 (0.79–1.21)
No. of observations	233	151	325	225
Waist circumference (cm)	1.03 (1.00–1.05)	1.03 (0.99–1.06)	1.00 (0.98–1.03)	0.99 (0.96–1.02)
No. of observations	233	151	323	224
Body fat percentage	1.01 (0.97–1.04)	1.00 (0.95–1.05)	0.98 (0.94–1.01)	0.98 (0.94–1.02)
No. of observations	233	151	321	222

a Adjusted for heavy physical work, driving motor vehicle, lifting heavy objects at work, previous musculoskeletal injury, socioeconomic status and education.

b Adjusted for heavy physical work, previous musculoskeletal injury, socioeconomic status and education.

ORs indicate relative change in odds for DD sum score equal to or greater than any given sum score when compared with all lower classes. In cases not violating the proportionality assumption, only the OR common to all sum score levels is shown. Otherwise, ORs are shown separately for each level of the outcome.

## Discussion

Waist circumference and two of the MRI-based obesity measurements, sagittal diameter and abdominal diameter, were associated with lumbar disc degeneration among males, while no such associations were found among females. Previously, BMI has been associated with disc degeneration in the same population among males, but not among females, which is in concordance with the current results (data not shown).

### Adiposity Measurements

Prior studies on body composition have used several methods for body composition, but no golden standard for this purpose exists. Underwater weighing was earlier considered the most accurate measure, but was later replaced by DXA and MRI [27]. Recently, MRI adiposity measurements have proved to be superior to WC in the assessment of visceral abdominal fat [28]. In our study, VST and DST were not associated with disc degeneration among males, whereas two other MRI-based obesity measures were. However, the usefulness of VST may have been underestimated in our study because VST was immeasurable in 43 participants. Subcutaneous adipose tissue measured though MRI on the back (similar to DST) is also believed to be a better total body adiposity estimator than WC or waist-to-hip ratio [29]. SAD on MRI has been considered a good risk estimator of cardiovascular diseases [30] and, moreover, SAD [28] and AD [31] are regarded as good measures of visceral adipose tissue. Fifty-three of the participants may have had even higher AD and SAD than estimated, which would strengthen the association of these measures with disc degeneration.

We found that the association of WC with disc degeneration was of the same magnitude as two of the MRI-based obesity measures. This implies that WC could be used clinically as a quick, low-cost measurement for evaluating abdominal adiposity as a risk factor of disc degeneration.

We used midsagittal MR images to diminish measurement errors. In the midsagittal images, no abdominal muscles distracted the measurements, as the ventral starting point was linea alba. Similarly, the dorsal endpoint for SAD was the subcutaneous fascia just beneath the spinous processes of the vertebrae. Thus the main compartments measured in SAD were subcutaneous fat, visceral fat, bowels, and bony spinal column.

The difference between females and males can partly be explained by the adipose tissue storage differences between genders. Most of the MRI adiposity measurements were performed at the abdominal level, which is the main fat storage area among males. Among females, the fat tissue is located mainly in the thighs and buttocks [32]. In this study, we did not measure gluteal adiposity thickness or thigh fat storage thickness, and we were not able to study the significance of high adiposity level *per se*, especially among females. The hormonal differences between genders may also have an effect. In addition, we had no exact data on the exposure times of adiposity. We had data on weight and height at seven years, but there was a nine-year gap before the next measurement at 16 years. However, our latest findings on an association of high BMI in early childhood with disc degeneration seem to be similar to the results of this study: the significant findings are among males only (data not shown).

### Possible Mechanisms Underlying the Association between Obesity and Disc Degeneration

Increased mechanical load on intervertebral discs due to abdominal obesity may explain our findings among males. Since fat tissue is usually located around the hip level in females, it does not mechanically load the lumbar spine to the same extent as it does in males [32]. Excessive mechanical loading will result in mechanical stresses within the disc tissue, which may ultimately lead to the loss of cell viability, altered biosynthesis of extracellular matrix and enzymes, and eventually matrix remodelling, similarly to disc degeneration [33]. Another mechanism is atherosclerosis, which has been related to disc degeneration in adult populations. The well-known risk factors for atherosclerosis are smoking, hypertension, high total cholesterol, high LDL cholesterol, high triglyceride, carotid intima-media thickness, and diabetes [11]. Obesity is a risk factor for such conditions. Moreover, obesity-induced chronic inflammation has also been associated with atherosclerosis [34], [35]. Obesity has been found to increase proinflammatory adipocytokines, which are produced in adipose tissue. These adipocytokines stimulate hepatocytes to produce inflammatory markers, especially C-reactive protein, in obese adult and adolescent individuals [34], [36], [37]. These findings suggest that overweight is a low-grade systemic inflammatory condition, which may cause endothelial dysfunction and subsequently atherosclerosis [34], [38]. Our study participants were young adults and it is doubtful that they had atherosclerosis severe enough to compromise lumbar disc blood supply and nutrition. However, overweight has also been associated with histological [39] and macroscopic [14], [16] disc degeneration. It can cause changes in disc structure and impair its healing process by decreasing metabolite transport into the disc [40].

### Strengths and Weaknesses of the Study

The strength of our study is its population-based birth cohort design, although some participation bias is acknowledged. The narrow age range makes it possible to minimize the confounding effect of age. Although the participants of the present study had slightly healthier lifestyles (non-smokers, less time spent sitting, physically more active, and leaner) and came from families with higher socioeconomic status slightly more often than the non-participants, a higher proportion of them had low back pain. Thus, the influence of these differences on the observed associations would be minimal [21]. An additional factor of interest was the higher proportion of missing data among non-participants compared to that of participants. The data was recorded at 16 years of age, when the participants did not yet know whether or not they would participate in the lumbar MRI study. We suppose that more meticulous personality traits among participants may have increased their willingness to participate and also partly explain the respective differences in lifestyles. However, we do not believe that this introduces any substantial bias to our results.

We analyzed the reliability of adiposity MRI measurement and found the ICC to be very good in each adiposity measurement. We used qualitative modified Pfirrmann classification [21] in the assessment of disc degeneration, which is considered inferior to the quantitative assessment of disc degeneration [41]. In our previous studies, we found good inter-rater reliability between the results found by a medical student trained to evaluate disc degeneration on MRI and the consensus reading of two experienced readers [21]. The inter-rater reliability for disc degeneration between two expert musculoskeletal radiologists was moderate to good at the three lowest levels. The kappa-values were lower than previously reported [42], but the disagreements were settled by consensus.

The main limitation of our study is the cross-sectional design of the imaging. Therefore, the onset and progression of disc degeneration in the lumbar spine among the study participants remains unknown. However, as disc degeneration has an early onset [13], [43], [44], a very large cohort starting in early teenage years with annual imaging for several decades would be needed to study the natural progression of degenerative changes and their association with unhealthy behaviors, which is beyond the scope of the present study. Moreover, due to the lack of longitudinal imaging data, we cannot rule out the possibility that reverse causation, i.e. presence of disc degeneration may result to reduced level of physical activity, which could contribute to weight gain.

### Disc Degeneration and Low Back Pain

LBP and disc degeneration have been associated with each other among both adolescents [13], [45] and adults [46]. We also found this association in the present study population. Severe low back symptoms over a three-year period were associated with disc degeneration, and the association was stronger for moderately degenerated discs than mildly degenerated ones [47]. However, the clinical relevance of disc degeneration is questioned by the fact that its prevalence is also high among asymptomatic participants [48], [49], although patterns of more severe degeneration are more likely to be associated with the severity of symptoms [46]. Despite this, based on a systematic review [12], we concluded that disc degeneration was significantly associated with LBP. Furthermore, studies have noted that the development of disc degeneration in early age may lead to severe disc degeneration early on, presenting a long-term risk of recurrent LBP [50]. As such, the study on disc degeneration has clinical relevance and warrants investigation to identify risk factors for preventative measures.

### Conclusions

Measures of abdominal obesity, sagittal diameter, abdominal diameter and waist circumference, were associated with disc degeneration among young adult males. Waist circumference can be used clinically to assess abdominal adiposity as a risk factor of disc degeneration. These factors should be taken into account when assessing the ‘risk profile’ of an individual’s development of disc degeneration.

## Supporting Information

Table S1 Comparison of participants and non-participants of the Oulu Back Study population at 16 years of age.
(DOC)

Table S2 Variables checked for confounding. Participants responded to these questions at 18 years of age (except socioeconomic status, at 16 years).
(DOC)
