# Supplementary material for: Association of Abdominal Obesity with Lumbar Disc Degeneration – A Magnetic Resonance Imaging Study
Source: PLoS One. 2013 Feb 13;8(2):e56244. doi: 10.1371/journal.pone.0056244 (PMC3571955; doi:10.1371/journal.pone.0056244)
Supplement: Table S1 — Comparison of participants and non-participants of the Oulu Back Study population at 16 years of age. (DOC) [file pone.0056244.s001.doc]

| Table S1. Comparison of participants and non-participants of the Oulu Back Study population at 16 years of age. | | | |
| --- | --- | --- | --- |
|  | Non-participants  (N=2408) | Participants  (N=561) | Whole study population  (N=2969) |
| Socioeconomic status | % (N) | | |
| Higher administrative | 33 (536) | 38 (198) | 34 (734) |
| Lower administrative | 21 (351) | 22 (113) | 22 (464) |
| Entrepreneur | 5 (87) | 4 (23) | 5 (110) |
| Employee | 36 (592) | 33 (170) | 35 (762) |
| Other | 4 (68) | 3 (17) | 4 (85) |
| All | 100 (1634) | 100 (521) | 100 (2155) |
| *Missing data* | *32 (774)* | *7 (40)* | *27 (814)* |
| Low back pain | % (N) | | |
| No | 60 (1034) | 54 (288) | 59 (1322) |
| Yes | 40 (684) | 46 (242) | 41 (926) |
| All | 100 (1718) | 100 (530) | 100 (2248) |
| *Missing data* | *29 (690)* | *6 (31)* | *24 (721)* |
| Smoking | % (N) | | |
| No^*^ | 85 (1452) | 91 (481) | 87 (1933) |
| Yes | 15 (253) | 9 (45) | 13 (298) |
| All | 100 (1705) | 100 (526) | 100 (2231) |
| *Missing data* | *29 (703)* | *6 (35)* | *25 (738)* |
| Gender | % (N) | | |
| Female | 47 (1121) | 58 (326) | 49 (1447) |
| Male | 53 (1287) | 42 (235) | 51 (1522) |
| All | 100 (2408) | 100 (561) | 100 (2969) |
| Physical activity^#^ |  | % (N) |  |
| Once a week or less | 39 (668) | 33 (175) | 37 (843) |
| 2–3 times a week | 31 (527) | 34 (182) | 31 (709) |
| At least 4 times a week | 31 (526) | 33 (176) | 31 (702) |
| All | 100 (1721) | 100 (533) | 100 (2254) |
| *Missing data* | *29 (687)* | *5 (28)* | *24 (715)* |
|  | Mean (N; %) | | |
| Sitting hours per week | 6.7 (1719; 71) | 6.1 (533; 95) | 6.6 (2252; 76) |
| Body mass index (kg/m^2^) | 21.1 (1619; 67) | 20.9 (545; 97) | 21.1 (2164; 73) |
| Waist circumference (cm) | 74.0 (1600; 66) | 72.7 (539; 96) | 73.7 (2139; 72) |
| ^*^Includes occasionally smoking participants  ^#^Participating in brisk exercise for at least twenty minutes outside school hours | | | |
