# Supplementary material for: Association of Abdominal Obesity with Lumbar Disc Degeneration – A Magnetic Resonance Imaging Study
Source: PLoS One. 2013 Feb 13;8(2):e56244. doi: 10.1371/journal.pone.0056244 (PMC3571955; doi:10.1371/journal.pone.0056244)
Supplement: Table S2 — Variables checked for confounding. Participants responded to these questions at 18 years of age (except socioeconomic status, at 16 years). (DOC) [file pone.0056244.s002.doc]

| Table S2. Variables checked for confounding. Participants responded to these questions at 18 years of age (except socioeconomic status, at 16 years). | | | | |
| --- | --- | --- | --- | --- |
| Variables included in the final adjusted model | |  | Variables not included in the final adjusted model | |
| Phrase used in text | Definition |  | Phrase used in text | Definition |
| Heavy physical work | Very strenuous work involving lifting or carrying heavy objects (digging, shovelling, hammering etc.) (yes/no) |  | Kneeling/squatting at work | Kneeling or squatting for ≥1 h/day (yes/no) |
| Driving a motor vehicle | Driving a car, tractor or other motor vehicle for ≥4 h/day for >3 months/year (yes/no) |  | Awkward trunk postures | Working standing or on one’s knees in a position leaning forward without support for ≥1 h/day (yes/no) |
| Lifting heavy objects at work | Manually lifting, carrying or pushing objects heavier than 20 kg ≥10 times/day (yes/no) |  | Standing or walking at work | Standing or walking for ≥5 h/day (yes/no) |
| Previous injury | Injuries other than a fracture requiring medical consultation at any of these sites: (1) Head, (2) Neck, (3) Shoulders, (4) Low back, (5) Elbows, (6) Wrists, forearms, (7) Knees, (8) Ankles, feet (yes/no) |  | Hands above shoulder level at work | Work with hands above shoulder level for ≥1 h/day (yes/no) |
| Socioeconomic status | Parents’ socioeconomic status: (1) Entrepreneur, (2) White-collar worker, (3) Blue-collar worker, (4) Laborer, (5) Other |  | Using vibrating tool(s) | Using vibrating tool(s) at work for ≥2 h/day (yes/no) |
| Student status | Current situation: (1) Not studying, (2) High school, (3) Vocational school, (4) Studying elsewhere |  | Participation in sports | (1) Never, (2) Occasionally,  (3) Regularly |
|  |  |  | Sedentary work | Sedentary work with limited walking (yes/no) |
|  |  |  | Lifting medium weight objects at work | Manually lifting, carrying or pushing objects weighing a maximum of 5 kg >2 times/min for ≥2 h/day (yes/no) |
